# The HSV1 Tail-Anchored Membrane Protein pUL34 Contains a Basic Motif That Supports Active Transport to the Inner Nuclear Membrane Prior to Formation of the Nuclear Egress Complex

**DOI:** 10.3390/v13081544

**Published:** 2021-08-05

**Authors:** Christina Funk, Débora Marques da Silveira e Santos, Melanie Ott, Verena Raschbichler, Susanne M. Bailer

**Affiliations:** 1 Fraunhofer Institute for Interfacial Engineering and Biotechnology IGB, 70569 Stuttgart, Germany; Christina.Funk@igb.fraunhofer.de (C.F.); deboramarquesss@hotmail.com (D.M.d.S.eS.); 2 Institute for Interfacial Engineering and Plasma Technology IGVP, University of Stuttgart, 70174 Stuttgart, Germany; 3 Max von Pettenkofer-Institute, Ludwig-Maximilians-University Munich, 80539 Munich, Germany; melanieott@gmx.de (M.O.); verenarasch@gmail.com (V.R.)

**Keywords:** HSV1, pUL34, pUL31, nuclear egress, NEC, targeting integral membrane proteins, TA membrane proteins, inner nuclear membrane (INM), nuclear import, importins

## Abstract

Herpes simplex virus type 1 nucleocapsids are released from the host nucleus by a budding process through the nuclear envelope called nuclear egress. Two viral proteins, the integral membrane proteins pUL34 and pUL31, form the nuclear egress complex at the inner nuclear membrane, which is critical for this process. The nuclear import of both proteins ensues separately from each other: pUL31 is actively imported through the central pore channel, while pUL34 is transported along the peripheral pore membrane. With this study, we identified a functional bipartite NLS between residues 178 and 194 of pUL34. pUL34 lacking its NLS is mislocalized to the TGN but retargeted to the ER upon insertion of the authentic NLS or a mimic NLS, independent of the insertion site. If co-expressed with pUL31, either of the pUL34-NLS variants is efficiently, although not completely, targeted to the nuclear rim where co-localization with pUL31 and membrane budding seem to occur, comparable to the wild-type. The viral mutant HSV1(17^+^)Lox-UL34-NLS mt is modestly attenuated but viable and associated with localization of pUL34-NLS mt to both the nuclear periphery and cytoplasm. We propose that targeting of pUL34 to the INM is facilitated by, but not dependent on, the presence of an NLS, thereby supporting NEC formation and viral replication.

## 1. Introduction

Herpesviral propagation is an intricate process initiated in the infected nucleus. Capsids formed in the nucleoplasm and packaged with one copy of the viral DNA are too large for export through the nuclear pore. To overcome the nuclear envelope barrier, herpesviruses have established a conserved membrane-budding process called nuclear egress. Primary envelopment occurs by association of nucleocapsids with the inner nuclear membrane (INM) and subsequent membrane budding resulting in enveloped nucleocapsids that transit the perinuclear space. Fusion of primary envelope with the outer nuclear membrane (ONM) allows for the de-envelopment of the nucleocapsids, further maturation in the cytoplasm and re-envelopment at the cytoplasmic membranes, resulting in infectious virions [1,2,3,4,5].

Two essential viral proteins, pUL34 and pUL31, with orthologs in all herpesviruses, form the nuclear egress complex (NEC), which is essential for this process [6,7,8,9]. Herpes simplex virus type 1 (HSV1) pUL34 represents an integral membrane protein featured by a cyto-/nucleoplasmically exposed N-terminal domain and a C-terminal transmembrane domain (TM) called the tail-anchor (TA) domain [10,11]. The nuclear phosphoprotein pUL31 consists of a variable N-terminal part enriched in basic residues ([12] and references therein) and a larger conserved C-terminal domain. Interaction of both NEC partners is mediated by an N-terminal domain of pUL34 (aa 137–181) together with the first of four conserved regions CR1 of pUL31 (for review see [6]). Both in vivo and in vitro, recombinantly expressed pUL31 and pUL34 are sufficient to induce the membrane-budding process, which results in vesicles with a highly ordered coat on the inside [13,14,15,16,17,18]. The NEC thus represents the minimal virus-encoded membrane-budding machinery that is sufficient to drive membrane budding and scission of vesicles during nuclear egress. Crystal structures of dimeric NECs of various orthologs revealed an elongated heterodimer that is organized and stabilized by two interfaces between pUL34 and pUL31 [15,19,20,21,22]. Formation of the NEC at the INM requires that both pUL31 and pUL34 are transported into the nucleus. While pUL31 harbors a bipartite nuclear localization sequence (NLS) that is redundant in the viral context and enters the nucleus by transport through the central nuclear pore [12], nuclear import of pUL34 is poorly understood.

The transport of integral membrane proteins to the INM is essential for various nuclear processes and thus crucial for the entire life cycle of eukaryotes. Numerous membrane proteins of the INM were identified that are involved in inheritable diseases (for review see [23]). Despite its importance, the targeting of integral membrane proteins to the INM is one of the least understood cellular transport processes. Essentially, two models are debated for transport of integral membrane proteins to the INM (as reviewed by [24]). First, there is the diffusion-retention model, where integral membrane proteins are inserted into the endoplasmic reticulum (ER) membrane and laterally diffuse to the INM, where retention occurs by INM residents; secondly, there is the transport factor-mediated model, where integral membrane proteins are actively transported to the INM using soluble transport factors of the importin α/β family ([25] and references therein). TA membrane proteins like pUL34 represent a particular kind of integral membrane proteins [10,11]. Due to their C-terminal transmembrane domain, TA membrane proteins are released from the ribosome to expose this domain for post-translational membrane insertion. For this reason, TA membrane proteins may be soluble for a short period of time, potentially providing them access to transport through the central pore channel and subsequent membrane insertion at the INM.

In order to gain further insight into transport of TA membrane proteins to the INM, we made use of the HSV1 infection as a functional reporter system. We identified a basic motif between residues 178 and 194 of pUL34 resembling a bipartite NLS. We demonstrate that this NLS is functional and important for the targeting of pUL34 to the nuclear rim, and most likely to the INM, and that it plays a role in viral replication. We propose that the pUL34-NLS targets pUL34 to the POM for efficient translocation to the INM prior to herpesviral NEC formation and capsid nuclear egress.

## 2. Materials and Methods

### 2.1. Cells, Viruses and General Methods

HeLa (ATCC-No. CCL-2), Hep2 (ATCC-No. CCl-23) and Vero cells (ATCC-No. CCL-81) were cultured in Dulbecco’s modified Eagle’s medium containing 10% FCS. The BAC-DNA pHSV1(17^+^)Lox (kindly provided by B. Sodeik) [26,27,28] and virus thereof (HSV1(17^+^)Lox) were used for all experiments. Plasmid transfection was done using Effectene Transfection Reagent (Qiagen GmbH, Hilden, Germany) according to manufacturer’s instructions. In contrast, BAC transfection was done using Lipofectamine 2000 (Thermo Fisher Scientific Inc., Waltham, MA, USA). HSV1 propagation, plaque formation, virus titration and kinetics [11,29], as well as the Y2H method [30], were described previously.

### 2.2. Plasmids

Plasmids were generated by classical restriction cloning, Gateway recombination cloning (Thermo Fisher Scientific Inc., Waltham, MA, USA) or Gibson assembly cloning (New England Biolabs Inc., Ipswich, UK) according to the manufacturer’s protocols. Single base-pair exchanges were introduced using the QuikChange site-directed mutagenesis kit (Agilent Technologies, Santa Clara, CA, USA) and verified by sequencing. The primers used to generate plasmids are listed in Table 1.

Constructs encoding pUL34 or mutants thereof were cloned into Gateway-compatible pCR3-C-HA, pCR3-C-MBP, pCDNA6.2-N-YFP, pCDNA6.2-C-YFP or pGBKT7 vectors. Truncation mutants of pUL34 as well as plasmids used for Y2H were generated using Gateway cloning as described previously [31]. Plasmid pCR3-N-Myc-UL31 as well as pEXPR-IBA5-UL34 expressing Strep-pUL34 were described previously [11]. Oligonucleotides used to generate pUL34-MBP-TM and different Strep-pUL34 mutants are listed in Table 1. The plasmid encoding EYFP-UL34-NLS was cloned based on EYFP-Nuc (Takara Bio Inc, Shiga, Japan), which was also used as a control. Plasmids pQE60-KPNA5, pQE60-KPNB1 and pQE32-RanQ69L were kindly provided by D. Görlich and U. Kutay, and pGPS-galK-kan was provided by Z. Ruzsics.

### 2.3. Immunofluorescence Microscopy

HeLa, Hep2 or Vero cells were grown on coverslips, transfected or infected and fixed with 2% formaldehyde/PBS for 15 min at room temperature (RT). Cells were permeabilized using 0.5% Triton X-100 (5 min, 4 °C). The binding of antibodies to the HSV1 Fc receptor-like proteins gE/gI was blocked with human IgG of seronegative individuals/PBS for at least 3 h at room temperature. Following incubation with primary antibodies (15 min, room temperature) and washing with PBS, the binding of anti-mouse, anti-rabbit or anti-chicken secondary fluorescently labelled antibodies (Thermo Fisher Scientific Inc., Waltham, MA, USA) was allowed (15 min, RT). Mouse monoclonal antibodies anti-HA (Thermo Fisher Scientific Inc., Waltham, MA, USA), anti-ICP0 (Santa Cruz Biotechnology, Dallas, TX, USA), anti-Lamin A/C (Santa Cruz Biotechnology, Dallas, TX, USA), anti-Myc (clone 9E10, kindly provided by J. von Einem) and anti-Strep tag II (IBA Lifesciences GmbH, Göttingen, Germany), as well as polyclonal rabbit antibodies anti-MBP (New England Biolabs Inc., Ipswich, MA, USA), anti-TGN46 (Bio-Rad, Hercules, CA, USA) and anti-chicken anti-pUL34 (kindly provided by R. Roller) (further blocking step using BlokHen^®^ solution (Aves Labs, Davis, CA, USA) for 30 min), were employed. Confocal laser scanning microscopes LSM710 (Zeiss, Oberkochen, Germany) and TCS SP5 (Leica, Mannheim, Germany) were used for sample examinations, and Zen-Lite (Zeiss, Oberkochen, Germany) and Adobe Photoshop (Adobe, San José, CA, USA) were used for processing of microscope recordings.

### 2.4. BAC Mutagenesis

The HSV1 UL34 mutants were generated using pHSV1(17^+^)Lox and a modified *galK* positive counterselection scheme essentially as described [11,32]. First, the UL34 coding region was replaced by a *galK*-kan cassette (Table 2), which was previously amplified using the pGPS-galK-kan plasmid (kindly provided by Z. Ruzsics) and primers equipped with 50 bp homologies flanking the UL34 locus (Table 1). In a second step, the *galK*-kan cassette was substituted with a UL34 region encoding UL34-NLS mt or wild-type UL34 (Table 2). Virus reconstitution was performed through transfection of Vero cells with the respective BAC-DNA using Lipofectamin 2000 (Thermo Fisher Scientific Inc., Waltham, MA, USA).

### 2.5. Formation of Nuclear Transport Complexes

All proteins used to analyze the formation of nuclear transport complexes were recombinantly expressed in suitable *E. coli* strains (JM101 (pQE60-KPNB1, pQE32-RanQ69L), BL21(DE3) (pET9d-GST-UL34ΔTM), M15/pREP4 (pQE60-KPNA5). Protein expression was induced by addition of 1 mM IPTG (culture with OD_600_ 0.6) for 4 h at RT. Bacterial lysates were generated in universal buffer (20 mM HEPES, 100 mM potassium acetate, 2 mM magnesium acetate, 0,1% Tween 20, 10% glycerin; pH 7.5), in the presence of protease inhibitors by mechanical disruption and subsequently cleared by centrifugation. Recombinantly expressed His- and GST-tagged proteins were affinity purified using Ni-NTA agarose (Qiagen GmbH, Hilden, Germany) and GSH sepharose (GE Healthcare, Chicago, IL, USA), respectively. Protein purification was performed in a universal buffer with the buffer additionally containing 20 mM of Imidazol in the case of His-tagged proteins. To allow for complex formation, the GST-tagged protein of interest was again bound to GSH sepharose followed by the addition of the His-tagged, putative binding partners. Immobilized proteins were released from the sepharose by incubation with 4x Lämmli buffer (15 min, RT) and analyzed by SDS-PAGE and Western blotting using goat anti-GST (Sigma-Aldrich, St. Louis, MO, USA) and mouse anti-His antibodies (Qiagen GmbH, Hilden, Germany), followed by POX-conjugated secondary reagents.

## 3. Results

### 3.1. Membrane Insertion of pUL34 Occurs Prior to Its Nuclear Import

**Figure 1:**
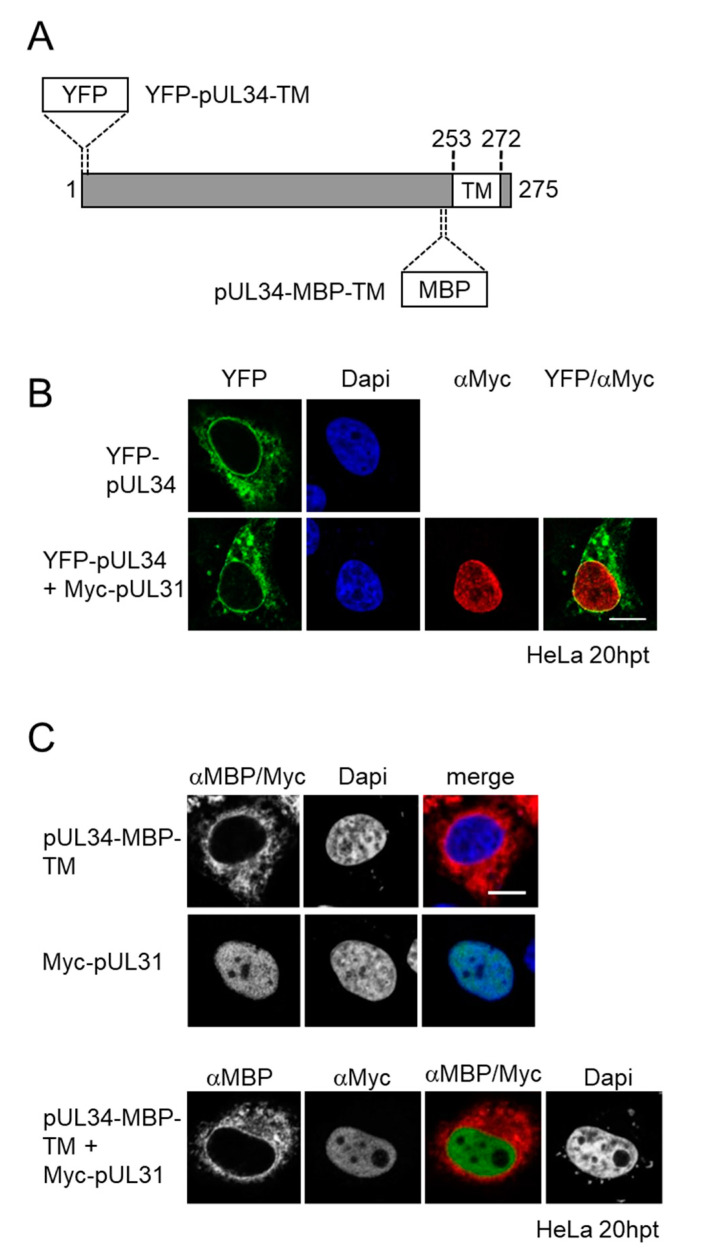
Membrane insertion of pUL34 occurs prior to its nuclear import. (**A**) Schematic depiction of the TA membrane protein pUL34 with the C-terminal transmembrane domain (TM) and either the N-terminal YFP tag (YFP-pUL34-TM) or a pre-TM-inserted MBP-tag (pUL34-MBP-TM); (**B**,**C**) HeLa cells were transfected with plasmids encoding YFP-pUL34-TM, pUL34-MBP-TM or Myc-pUL31 either alone ((**B**,**C**) top panels) or in combinations ((**B**,**C**), bottom panels). pUL31 localization was determined by using anti-Myc antibodies, while pUL34-MBP-TM was detected by anti-MBP antibodies followed by secondary reagents. YFP-tagged pUL34 was visualized directly using confocal microscopy. Nuclei were visualized by Dapi. The scale bar corresponds to 10 µm.

Previous data suggested that herpes simplex virus 1 (HSV1) pUL34 and pUL31 enter the nucleus separate of each other [12]. Full-length pUL34 carrying an N-terminal MBP tag, thereby exposing a cyto-/nucleoplasmic domain of about 75 kDa, was detected at ER-like structures and at the nuclear periphery irrespective of the presence of its NEC partner Myc-pUL31, the latter of which accumulated in the nucleus [12]. To corroborate and extend these findings, the cyto-/nucleoplasmic domain of pUL34 was N-terminally fused to YFP, resulting in a smaller cyto-/nucleoplasmic mass of about 60 kDa (Figure 1A). Again YFP-pUL34 was detected at ER-like structures and at the nuclear periphery (Figure 1B). In the presence of Myc-pUL31, the subcellular distribution of YFP-pUL34 resembled that of MBP-pUL34 (Figure 1B; [12]).

To exclude the interference of N-terminal tagging of pUL34 with its structural integrity potentially having an influence on its interaction with pUL31, an MBP-tag was inserted between residues 235 and 236 prior to the pUL34-TM domain, representing an unstructured region (Figure 1A,C). The transient expression of pUL34-MBP-TM, again exposing a cyto-/nucleoplasmic domain of about 75 kDa, resulted in a similar ER-like distribution as observed with YFP- and MBP-pUL34. In presence of Myc-pUL31, the subcellular distribution of pUL34-MBP-TM was unaltered, and both proteins located to separate subcellular entities. Together, these data indicate that pUL34 and pUL31 do not interact in the cytoplasm and that the membrane-anchored pUL34 needs to access a nuclear transport mode different from pUL31.

### 3.2. The NEC Protein pUL34 Contains a Functional Bipartite Nuclear Localization Sequence

**Figure 2:**
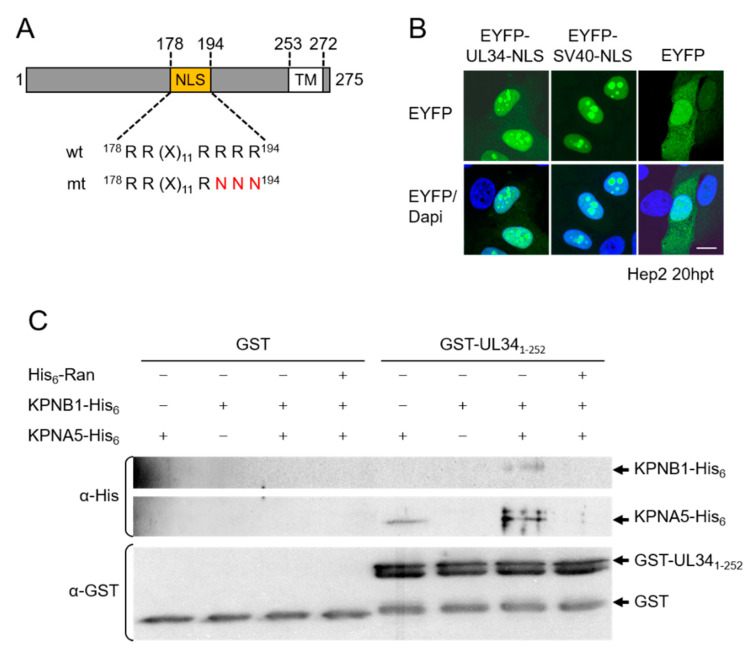
The NEC protein pUL34 contains a functional bipartite NLS. (**A**) Schematic depiction of the TA membrane protein pUL34 with the bioinformatically predicted bipartite NLS (residues 178–194) and the exchanged residues in case of the NLS mutation (red). (**B**) To determine whether the UL34-NLS can mediate the nuclear import of a cytoplasmic protein, Hep2 cells were transfected with plasmids encoding EYFP-UL34-NLS and EYFP-SV40-NLS as the positive control and EYFP alone as the negative control. Then, 20 h post transfection (hpt), EYFP was visualized directly and nuclei were detected by Dapi. Scale bar corresponds to 10 µm. (**C**) Formation of a nuclear import complex in vitro was started with binding of GST-pUL34_1-252_ or GST alone as the negative control to GSH sepharose followed by incubation with KPNB1-His_6_, KPNA5-His_6_ and His_6_-RanGTP, either alone or in combinations. Eluates were analyzed by SDS-PAGE followed by Western blotting using mouse anti-His or goat anti-GST antibodies, followed by POX-conjugated secondary reagents.

The finding that pUL34 reaches the nucleus independent of pUL31 suggested it contains its own import mechanism. Using bioinformatic tools, a basic motif was identified within the TA membrane protein pUL34, composed of RR(X)_11_RRRR, resembling a classical bipartite NLS (http://www.expasy.org/; Figure 2A). To be classified as a functional NLS, a given sequence has to fulfil several criteria, e.g., to trigger the nuclear import of an unrelated, cytoplasmic protein, and to mediate physical interaction with transport factors of the importin α/β family [33].

EYFP-pUL34-NLS, where pUL34 residues 178–194 (Figure 2A) were fused to EYFP, strongly accumulated in the nucleus (and nucleolus) comparable to EYFP-SV40-NLS containing the classical SV40-NLS, while EYFP lacking an NLS was distributed between the cytoplasm and nucleus (Figure 2B). Thus, residues 178–194 of pUL34 represent a potent NLS. The yeast two-hybrid (Y2H) assay revealed that pUL34_1-252_ redundantly interacted with α importins KPNA2, 4 and 5. pUL34_1-203_, which still contained the complete NLS, also interacted with all tested importins, while pUL34_1-180_ contained only the first half of the bipartite NLS, and pUL34_1-168_, where the complete NLS was deleted, showed no interaction. Finally, pUL34_1-252_-NLS mt, where three positively charged residues within one half of the predicted bipartite NLS were replaced by alanines (Figure 2A), also lost the ability to bind α importins. pUL34_1-252_ was able to form a trimeric complex with α importin KPNA5 and β importin KPNB1 (Figure 2C), and complex formation was inhibited in the presence of RanGTP, demonstrating the functionality of the pUL34-NLS (Figure 2C).

To analyze whether the identified classical NLS was active within the pUL34 context, the soluble cyto-/nucleoplasmic domain of pUL34 of about 27 kDa was C-terminally tagged and expressed as wild-type or NLS mutant. Indirect immunofluorescence analysis revealed that both pUL34_1-252_-HA and pUL34_1-252_-NLS mt-HA were exclusively located in the nucleoplasm, reflecting the ability of small proteins to enter the nucleus by diffusion [34]. Interestingly, in either case, soluble pUL34 showed an almost exclusive nuclear localization pointing to an undefined nuclear retention mechanism. pUL34_1-252_-MB, P where the HA-tag was replaced by MBP resulting in a protein of about 75 kDa, efficiently accumulated in the nucleus, while a residual protein was found in the cytoplasm. In contrast, pUL34_1-252_-NLS mt-MBP was exclusively detected in the cytoplasm and thus unable to enter the nucleus. Thus, the NLS of pUL34_1-252_ is able to mediate active transport in its protein context. In summary, we conclude that residues 178–194 of the NEC protein pUL34 compose a functional bipartite NLS.

### 3.3. Targeting of pUL34 to the Nuclear Rim Combines Active Transport and Retention by Its NEC Partner pUL31

**Figure 3:**
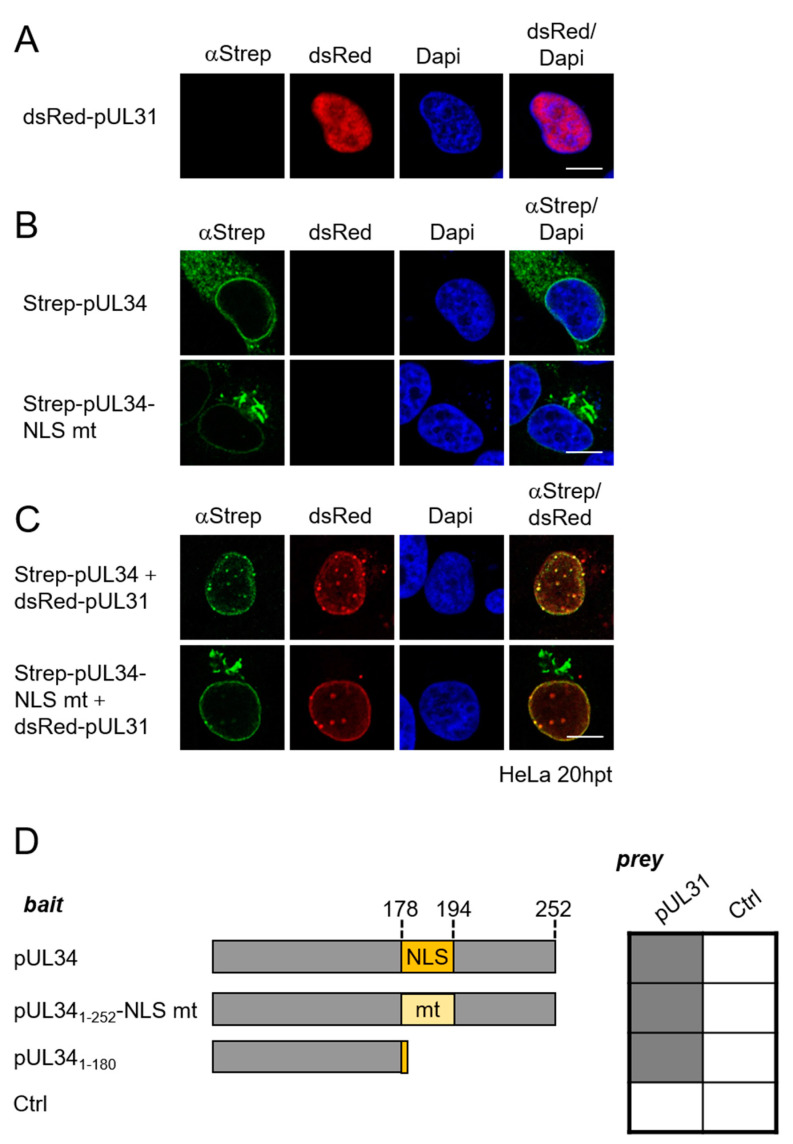
Targeting of pUL34 to the nuclear rim combines active transport and retention by its NEC partner pUL31. To determine the importance of the pUL34-NLS for subcellular localization of pUL34 and NEC formation, (**A**) dsRed-pUL31 and (**B**) Strep-UL34 or Strep-pUL34-NLS mt were expressed in HeLa cells for 20 h either (**A**,**B**) alone or (**C**) in combinations. Indirect immunofluorescence was performed using anti-Strep tag II antibodies followed by Alexa Fluor 488-conjugated secondary reagents. dsRed-pUL31 was visualized directly and nuclei were detected by Dapi. Scale bars correspond to 10 µm. (**D**) To determine whether mutagenesis of the pUL34-NLS plays a role in interaction of pUL34 with pUL31, the bait plasmids pUL34_1-252_, pUL34_1-252_-NLS mt and pUL34_1-180_ (schematically depicted on the left) were co-expressed with the pUL31 prey plasmid. Negative controls (Ctrl) were represented by the respective empty plasmids. Interaction was analyzed by Y2H using the HIS3 reporter gene activation. Grey squares display positive results and white squares display negative results.

To determine the relevance of the pUL34-NLS for the targeting of the full-length protein to the nuclear rim, the subcellular distribution of Strep-pUL34 was compared to the NLS mutant Strep-pUL34-NLS mt in the absence and presence of its NEC partner pUL31. In the absence of pUL31, wild-type Strep-pUL34 was localized to the ER and the nuclear periphery (Figure 3B). In contrast, the pUL34-NLS mt was primarily found at Golgi-like structures (Figure 3B). Upon co-expression with dsRed-pUL31, that alone was located to the nucleoplasm (Figure 3A), and wild-type pUL34 was efficiently recruited to the nuclear rim (Figure 3C), consistent with previous results [12,35,36]. If co-expressed with dsRed-pUL31, pUL34-NLS mt is recruited to the nuclear periphery with some residual localization of pUL34-NLS mt to Golgi-like structures. In either case, however, intense co-localizations of pUL34 and pUL31 resembling vesicular structures were observed at the nuclear periphery, consistent with NEC formation and budding activity (Figure 3C).

The NLS (residues 178–194) is located in close vicinity to the pUL31 interaction domain of pUL34. To determine whether mutagenesis of the NLS potentially affects pUL34 and pUL31 interaction, the Y2H system was applied. The bait plasmids expressing pUL34_1-252_, pUL34_1-252_-NLS mt or pUL34_1-180_ were co-transformed with a prey plasmid expressing pUL31 or a negative control. As shown in Figure 3D, either version of pUL34, whether containing an intact or a truncated NLS, was able to interact with pUL31 consistent with the pUL34 domain (residues 1–180), which lacks the sufficient NLS for pUL31 interaction [7,9]. We thus conclude that the interaction of pUL34 and pUL31, and thus NEC formation, is independent of the pUL34 region where the NLS is located and the NLS itself. Together, these data show that the NLS contributes to the efficient and directed targeting of pUL34 to the nucleus, while it is not required for retention by its NEC partner pUL31.

### 3.4. The pUL34-NLS Functions Independently of Its Position and Can Be Replaced by an SV40-NLS

**Figure 4:**
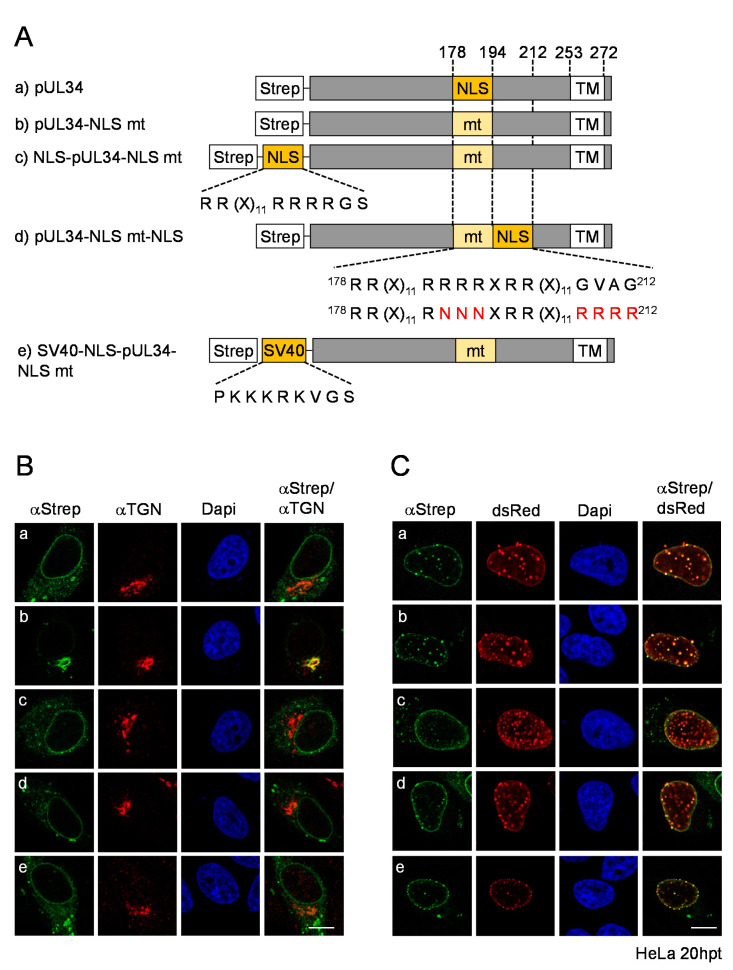
The pUL34-NLS functions independent of its position and can be replaced by an SV40-NLS. (**A**) Graphical depiction of Strep-pUL34, where the pUL34-NLS was replaced by the authentic NLS or a mimic in various positions. (**B**,**C**): (**a**) Strep-UL34, (**b**) Strep-pUL34-NLS mt, (**c**) Strep-NLS-pUL34-NLS mt and (**d**) Strep-pUL34-NLS mt-NLS as well as (**e**) SV40-NLS-pUL34-NLS mt were expressed in HeLa cells for 20 h either (**B**) alone or (**C**) in combination with dsRed-pUL31. Indirect immunofluorescence was performed using anti-Strep tag II and anti-TGN46 antibodies followed by Alexa Fluor 488- and 555-conjugated secondary reagents. dsRed-pUL31 was visualized directly and nuclei were detected by Dapi. Scale bars correspond to 10 µm.

The position of the NLS within pUL34 could be relevant for the NLS function. To analyze the impact of the NLS position on the subcellular localization of pUL34, the pUL34-NLS mt was further engineered by fusion of the authentic NLS to its N-terminal end, resulting in NLS-pUL34-NLS mt (Figure 4A). In addition, pUL34-NLS mt-NLS was generated, where an NLS mimicking the authentic pUL34-NLS was inserted C-terminally to the mutated one (Figure 4A). These mutants were transiently expressed and analyzed regarding their subcellular distribution. In contrast to pUL34-NLS mt, which perfectly co-localized with the TGN marker TGN46, both pUL34-NLS mt versions containing an additional NLS (NLS-pUL34-NLS mt and pUL34-NLS mt-NLS) were located at the nuclear envelope and the ER comparable to wild-type pUL34 (Figure 4B), indicating that an additional authentic or unrelated NLS can restore its localization to the ER. If co-expressed with dsRed-pUL31, either of the pUL34-NLS mutants was recruited to the nuclear rim. Strikingly, intense vesicular co-localizations of pUL34 with pUL31 in the nuclear periphery were observed in all pUL34-NLS variants, indicating that neither mutagenesis of the authentic NLS nor insertion of compensatory NLSs irrespective of their site seemed to abrogate NEC formation and budding (Figure 4C). In summary, the pUL34-NLS is required for efficient localization of pUL34 to the ER and the nuclear periphery, a function that is independent of the position and context of the NLS.

**Figure 5:**
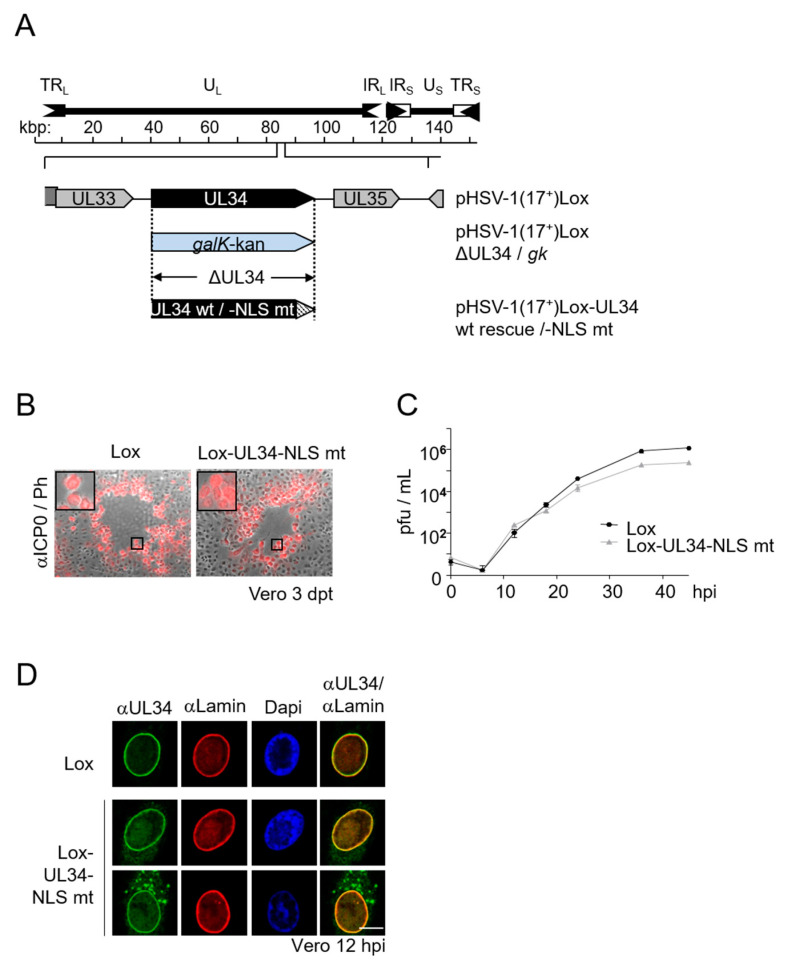
pHSV1(17^+^)Lox-UL34-NLS mt is modestly compromised in viral replication. (**A**) Schematic depiction of the pHSV1(17^+^)Lox genome, the localization of UL34 within the genome and the strategy for BAC mutagenesis. During BAC mutagenesis, UL34 is replaced by the *galK*-kan selection cassette (pHSV1(17^+^)Lox-ΔUL34/*gK*), followed by replacement of this cassette by the wild-type gene UL34 to reach pHSV1(17^+^)Lox-UL34 wt rescue, or by UL34 harboring the NLS mutation to generate pHSV1(17^+^)Lox-UL34-NLS mt. (**B**) To analyze virus propagation and plaque formation, Vero cells were transfected with BAC-DNA pHSV1(17^+^)Lox and pHSV1(17^+^)Lox-UL34-NLS mt, fixed 3 dpt and examined by indirect immunofluorescence using monoclonal anti-ICP0 antibodies followed by Alexa Fluor 595-labelled secondary antibodies. Black squares represent magnified sections. (**C**) Growth properties of Lox-UL34-NLS mt compared to Lox were determined by infection of Vero cells with an MOI of 0.1 in triplicates, the harvest of the cell culture supernatant at a different hpi and the titration on Vero cells using plaque assay. Graphical depiction includes error bars, and the statistical analysis of the time points t12, t24, t36 and t45 showed that the growth difference between wt and mt is significant with a *p*-value < 0.05. (**D**) To determine the pUL34-NLS mt localization in the viral context, Vero cells were infected at an MOI of 1 with Lox and Lox-UL34-NLS mt and analyzed at 12 hpi by IF using a combination of monoclonal anti-Lamin A/C with anti-pUL34 antibodies, followed by Alexa Fluor 555- and Alexa Fluor 488-conjugated secondary reagents. Nuclei were visualized by DAPI staining and confocal microscopy was applied for analysis. The scale bar corresponds to 10 μm.

The authentic bipartite NLS of pUL34 is largely composed of arginines. To compare the authentic NLS of pUL34 to that of SV40-NLS, a classical monopartite NLS enriched in lysins, the SV40-NLS-pUL34-NLS mt was generated, transiently expressed and analyzed for its subcellular distribution (Figure 4B,C). Upon isolated expression, SV40-NLS-pUL34-NLS mt was found at the nuclear envelope as well as at cytoplasmic ER-like structures similar to wild-type pUL34 and in contrast to pUL34-NLS mt (Figure 5B). Thus, a classical SV40-NLS can replace the authentic NLS of pUL34. In the presence of dsRed-pUL31, SV40-NLS-pUL34-NLS mt was recruited to the nuclear rim (Figure 4C), as seen for all pUL34-NLS mutants (Figure 4C). To conclude, the authentic bipartite pUL34-NLS composed of arginines can be substituted by a monopartite classical NLS without altering the subcellular distribution of pUL34 in the presence or absence of pUL31. Furthermore, intense vesicular co-localizations of pUL34 with pUL31 were observed both in the absence and presence of the pUL34-NLS and potential phosphosites therein [37,38], consistent with budding activity and a specific role of this motif in the transport and targeting of pUL34 distinct from the NEC formation and function.

### 3.5. Viral Replication Is Modestly Compromised in Absence of the pUL34-NLS

To determine the importance of the pUL34-NLS for viral replication, the UL34 locus of pHSV1(17^+^)Lox was replaced by the *galK*-kan selection cassette, which, in turn, was replaced either by wild-type UL34, resulting in pHSV1(17^+^)Lox-UL34 wt rescue (Lox), or by UL34-NLS mt, resulting in pHSV1(17^+^)Lox-UL34-NLS mt (Lox-UL34-NLS mt; Figure 2A, Figure 5A). Viral propagation of the mutant virus compared to the wild-type virus was investigated based on plaque formation as well as growth kinetics. Lox as well as Lox-UL34-NLS mt revealed comparable plaque sizes observed three days after the transfection of Vero cells with the respective BAC-DNA (Figure 5B). A detailed analysis of growth kinetics revealed that Lox-UL34-NLS mt showed 36–48 hpi when the virus yield plateaued, and about half a log difference in virus yield compared to Lox was observed, indicating a modest impairment in virus replication (Figure 5C).

To analyze the subcellular distribution of pUL34-NLS mt during infection, Vero cells were infected with Lox or Lox-UL34-NLS mt at an MOI of 1 and analyzed at 12 hpi by indirect immunofluorescence using a combination of anti-Lamin A/C with anti-pUL34 antibodies (Figure 5D). In Lox-infected cells, pUL34 perfectly co-localized with Lamin A/C in the nuclear periphery. In contrast, in Lox-UL34-NLS mt-infected cells, pUL34-NLS mt revealed two different localization patterns, either at the nuclear periphery comparable to the wild-type pUL34, or at both the nuclear periphery and ER-like structures, indicating an incomplete recruitment to the nuclear rim during infection.

Taken together, the mutation of pUL34-NLS results in a viable although attenuated virus associated with an incomplete or delayed transport of pUL34 to the nuclear periphery, potentially causing a delay in capsid nuclear egress.

## 4. Discussion

HSV1 infection is an attractive model system to analyze the transport of integral membrane proteins to the INM for a function essential to viral propagation. There, progeny nucleocapsids assembled in the nuclear interior, associated with the INM, where a membrane-budding process called nuclear egress is required for their release to the cytoplasm and eventually to the cell surface. Two essential and conserved viral proteins, the TA membrane protein pUL34 and the nucleo-phosphoprotein pUL31, form the NEC at the INM required for release of capsids from the nucleus. Recent data showed that pUL31 is actively imported through the central nuclear pore channel [12]. With this study using several pUL34 constructs containing differently positioned tags to enlarge the cyto-/nucleoplasmic domain, we found that pUL34 and pUL31 do not interact in the cytoplasm. Furthermore, our data strongly support that membrane insertion of pUL34 already occurs at the cytoplasmic membranes prior to nuclear import. Together, these data provide strong evidence that pUL34 and pUL31 take separate routes to the nucleus, where pUL31 uses the central pore channel, while pUL34 is transported along the POM.

Bioinformatic analysis of HSV1 pUL34 revealed two clusters of positively charged residues composed of RR and RRRRR within residues 178–194, which resemble a bipartite NLS. We show here that this sequence is able and sufficient to target an unrelated soluble cytoplasmic protein to the nucleus and that it forms a functional nuclear import complex with transport factors of the importin α/β family, fulfilling the criteria of a functional NLS [33]. Physical interaction of pUL34 with various α importins required both clusters of basic residues. Consistent with previous reports [11,39], transiently expressed pUL34_1-252_ lacking its transmembrane domain accumulated in the nucleus, a process that required a functional NLS, particularly if the protein was enlarged by fusion to MBP. Together, our data show that pUL34 contains a functional bipartite NLS that provides access to the classical nuclear import system.

Further analysis of this NLS revealed its impact on the subcellular distribution of a full-length TA membrane protein. Unlike the wild-type pUL34, which upon transient expression primarily locates to ER-like membranes, pUL34-NLS mt was predominantly located at the TGN. pUL34-NLS mt was redirected to the ER upon insertion of the authentic or a mimic NLS at different sites of pUL34, irrespective of the NLS sequence and position within pUL34. In the presence of its NEC partner pUL31, however, all variants of pUL34 were efficiently, although not completely, recruited to the nuclear rim. Together, these data indicate that the trafficking of pUL34 to the INM combines both an active NLS-dependent transport mechanism and retention at the INM by its NEC partner pUL31.

Different models have been proposed for transport of integral membrane proteins to the INM, including the diffusion-retention model and the transport factor-mediated model ([25] and references therein). Peripheral NPC channels are thought to be ~10 nm in width limiting the nucleoplasmic domain of an INM protein to ~60 kDa. A complex formed between the pUL34 domain of 27 kDa and transport factors of the importin α/β family would clearly exceed the peripheral pore channel. To overcome these limitations, a partial unfolding of the respective proteins is required, allowing them to stretch through the core NPC and expose their NLS to the central channel, thereby enabling the NLS to bind to transport receptors. Since the NLS of pUL34 is located at the C-terminal end of the cyto-/nucleoplasmic domain and in close vicinity to the peripheral pore membrane, unfolding is unlikely to expose the NLS to the pore channel. Thus, the underlying mechanism for active and classical transport of integral membrane proteins may be more complex, requiring further detailed studies.

Remarkably, a mutant virus encoding pUL34-NLS mt was replicating but modestly attenuated. Thus, while the NLS of pUL34 is not essential for viral replication, its presence seems to promote HSV1 growth. Interestingly, this viral phenotype correlates with partial mislocalization of pUL34-NLS mt to the cytoplasmic membranes during infection, suggesting an incomplete transport of pUL34-NLS mt to the nuclear periphery and likely to the INM, potentially explaining the attenuated phenotype. The importance of the NLS-dependent nuclear import could vary depending on the highly complex situation during infection, e.g., regarding expression and nuclear import dynamics of pUL31, or the availability of soluble transport factors. Thus, depending on the cellular and viral context, an NLS within pUL34 may be of advantage to viral replication and thus viral fitness. Interestingly, not all pUL34 orthologs seem to contain an NLS [40]. In addition to HSV1 pUL34, clusters of basic residues predicted to serve as NLS were identified in pUL34 orthologs of HSV2, HHV7, MCMV and EBV but were lacking in others [40]. This clearly indicates that the NLS is not a conserved feature of pUL34 and thus unlikely to be essential for nuclear egress and viral replication. This notion is supported by recent structural and functional data supported by the present study, where pUL34 lacking the second half of the pUL34-NLS forms stable NEC complexes [14,19]. Interestingly, while exposed in the cytoplasm, the pUL34-NLS may be partially masked by the binding of pUL31 upon NEC formation in the nucleus, making the NLS inaccessible for transport factors and finalizing pUL34 recruitment to the INM. Overall, it is thus conceivable that this NLS within pUL34 may promote the recruitment of pUL34 to the INM and thus NEC formation and capsid nuclear egress, depending on the dynamics of viral infection. This is particularly interesting in light of the capsid escort model, where pUL31 associates with nucleocapsids already in the nucleoplasm prior to escorting them to sites of primary envelopment [6,12,41]. In this scenario, part of the pUL34 molecules may actively reach the INM, there awaiting the pUL31 escorting the capsid from the nuclear interior to the INM. In parallel, pUL31 may readily interact with integral pUL34 to form NEC seeds, thereby retaining pUL34 at the INM. Thus, during HSV1 infection, both mechanisms of the active NLS-mediated import of pUL34 and retention by pUL31 may cooperate for a concerted nuclear egress, together promoting viral replication.

TA membrane proteins are a diverse group of eukaryotic membrane proteins targeted to various subcellular membranes, particularly to membranes of the secretory pathway and the nuclear envelope, among others. HSV1 encodes three TA membrane proteins, pUL34, pUL56 and pUS9. While pUL34 is located to the ER if expressed alone, pUL56 and pUS9 are targeted to the TGN [10], similar to pUL34-NLS mt, as observed in this study. Interestingly, unlike pUL34, pUL56 and pUS9 do not contain an active NLS. This could indicate that following post-translational insertion into ER membranes: TA membrane proteins are transported to the TGN by default and the mechanisms for retention or redirection to the ER are favorable for targeting to the nuclear periphery. Interestingly, the pUL34 mutant of pseudorabies (PRV) lacks an NLS [40] but contains the motif RQR 173–175 proposed to act as a Golgi retrieval signal. Mutagenesis to RQG results in the mislocalization of PRV pUL34 to the Golgi ([1] and references therein), resembling the subcellular distribution of HSV1-pUL34 lacking an NLS (Figure 3 and Figure 4). Since either the HSV1 or PRV pUL34 mutant is attenuated in viral replication, the ER may represent a favorable starting point for transport to the INM, since it is continuous with the ONM, POM and INM. At present, the mechanisms of such a retrieval or selection process for transport to the INM is unclear. However, together these data may indicate that different mechanisms exist, one of which involves classical transport factors. For further analysis, HSV1 could serve as a functional reporter system to gain insight into a protein transport process that is vital for nuclear functions far beyond herpesviral infections.

## 5. Conclusions

- The nuclear import of the HSV1 NEC partners pUL34 and pUL31 ensues separately from each other.
- HSV1 pUL34 contains a functional bipartite NLS that mediates the physical interaction with transport factors of the importin α/β family and the nuclear import of an unrelated cytoplasmic protein.
- In the absence of its NLS, pUL34 is mislocalized to the TGN but retargeted to the authentic localization at the ER upon the insertion of the authentic or a mimic NLS, independent of the insertion site.
- If co-expressed with pUL31, the pUL34-NLS mt is efficiently, although not completely, targeted to the nuclear rim, where NEC formation and membrane budding seemed to occur.
- A viral mutant expressing pUL34-NLS mt is modestly attenuated but viably associated with the partial mislocalization of pUL34-NLS mt in the cytoplasm.
- HSV1 pUL34 reaches the nuclear rim and most likely the INM by an NLS-dependent active process combined with retention by its NEC partner pUL31, thereby promoting viral replication.

## Figures and Tables

**Table 1:** Primer sequences used for plasmid cloning, site-directed mutagenesis and BAC mutagenesis.

No.	Name	Sequence	Use
1	UL34_vec_fw	GCAGACTGGAAGCTGGCACCAGGCCCTGCGG	Gibson
2	UL34_vec_rv	TGCTTCCCGGGCGGGAGGGCCCTTGGGTTAAC	Gibson
3	MBP_fw	TCCCGCCCGGGAAGCAAAATCGAAGAAGGTAAA	Gibson
4	MBP_rev	CCTGGTGCCAGCTTCCAGTCTGCGCGTCTTTCAGG	Gibson
5	UL34-NLS-mt_fw	CGCCGAGCAGGCTATTACCCGTAACAACAACACCCGGCGGTCCCGGGG	mutagenesis
6	UL34-NLS-mt_rev	CCCCGGGACCGCCGGGTGTTGTTGTTACGGGTAATAGCCTGCTCGGCG	mutagenesis
7	NLS-UL34-NLS-mt_vector_fw	GCGGGACTGGGCAAGCCCTACAC	Gibson
8	NLS-UL34-NLS-mt_vector_rev	CCTCGAGGGATCCCCGGGTACCGAG	Gibson
9	NLS-UL34-NLS-mt_fw	CCGGGGATCCCTCGAGGAGACGGATCCTGTGC	Gibson
10	NLS-UL34-NLS-mt_rev1	AGTCCCGCGGATCCTCGGCGGCGACGGGTAATAG	Gibson
11	NLS-UL34-NLS-mt_rev2	TGTAGGGCTTGCCCAGTCCCGCGGATCCTC	Gibson
12	UL34-NLS-mt-NLS_fw	CCGAGGCCGGGCTGCGGCGGCGCCGAACGGGTTTC	mutagenesis
13	UL34-NLS-mt-NLS_rev	CGGAAACCCGTTCGGCGCCGCCGCAGCCCGGCCTC	mutagenesis
14	SV40NLS-UL34-NLS-mt_fw1	GCGGTCCCGAATTCGAGCTCGGTAC	Gibson
15	SV40NLS-UL34-NLS-mt_rev1	CCTACCTTTCTCTTCTTTTTTGGCCTCGAGGGATCCC	Gibson
16	SV40NLS-UL34-NLS-mt_fw2	GCCGAGACCGCGGTCCCGAATTCGAG	Gibson
17	SV40NLS-UL34-NLS-mt_rev2	CTTGCCCAGTCCCGCGGATCCTACCTTTCTCTTCTTTTTTG	Gibson
18	EYFP-UL34-NLS_fw	TCAGATCCGCTAGCGCTACCGGTCGCCACCATGGTGAGCAAGGGCGAG	restriction cloning
19	EYFP-UL34-NLS_rev1	CTGCTCGGCGGCGCGGCACAGAATCCGTCTCGTAGCTCGAGATCTGAG	restriction cloning
20	EYFP-UL34-NLS_rev2	TTTGGATCCTAAGGTTCGGCGGCGACGGGTAATAGCCTGCTCGGCGGCGC	restriction cloning
21	UL34/gk_fw	GAACCCTTTGGTGGGTTTACGCGGGCACGCACGCTCCCATCGCGGGCGCCCCTGTTGACAATTAATCATCGGCA	BAC
22	UL34/gk_rev	GCGAAGGCGTCCGGAACGCACTGGCGATTAGGGCGGCGGTGCGTCCTTTTGCCAGTGTTACAACCAATTAACC	BAC
23	UL34_fw	GAACCCTTTGGTGGGTTTACGCGGGCACGCACGCTCCCATCGCGGGCGCCATGGCGGGACTGGGCAAGCCCTAC	BAC
24	UL34_rev	GCGAAGGCGTCCGGAACGCACTGGCGATTAGGGCGGCGGTGCGTCCTTTTTTATAGGCGCGCGCCAGCACCAAC	BAC

**Table 2:** BAC mutagenesis.

No.	BAC	Inserted Fragment	Recipient BAC
1	pHSV1(17^+^)Lox		
2	pHSV1(17^+^)Lox ΔUL34/gK	*galK*-kan in UL34 locus (bp 1-825); PCR with oligos no. 17 and 18 and pGPS-*galK*-kan as template	No. 1
3	pHSV1(17^+^)Lox-UL34 wt rescue	UL34 wt; PCR with oligos no. 19 and 20 and pDONR207-UL34_1-275_ as template	No. 2
4	pHSV1(17^+^)Lox-UL34-NLS mt	UL34-NLS mt; PCR with no. 19 and 20 and pDONR207-UL34_1-275_-NLS mt as template	No. 2

## Data Availability

Not applicable.

## Abbreviations

BAC	bacterial artificial chromosome
ER	endoplasmic reticulum
HSV1	Herpes simplex virus type 1
INM	inner nuclear membrane
MBP	Maltose binding protein
NEC	nuclear egress complex
NLS	nuclear localization sequence
ONM	outer nuclear membrane
POM	pore membrane
TA	tail-anchor
TGN	trans Golgi network
Y2H	yeast two-hybrid
YFP	yellow fluorescent protein
